# A Systematic Review of the Utility of Residual Vein Obstruction Studies in Primary and Secondary Venous Thrombosis

**DOI:** 10.1155/2013/247913

**Published:** 2013-11-19

**Authors:** Murali Janakiram, Matthew Sullivan, Marina Shcherba, Shuang Guo, Henny H. Billett

**Affiliations:** ^1^Division of Hematology, Montefiore Medical Center and the Albert Einstein College of Medicine, 3411 Wayne Avenue, Ground Floor, Bronx, NY 10467, USA; ^2^Department of Medicine, Montefiore Medical Center and the Albert Einstein College of Medicine, 3411 Wayne Avenue, Ground Floor, Bronx, NY 10467, USA

## Abstract

*Background*. Residual vein obstruction (RVO), the persistence of venous thrombosis with time and often after anticoagulation, may indicate a systemic prothrombotic condition. Prior studies have shown varying efficacy in using RVO as a risk factor for future venous thromboembolic (VTE) recurrence. *Methods*. To assess whether positive RVO imaging predicts recurrent VTE events, we performed a meta-analysis on studies in which patients with documented VTEs, anticoagulated for a minimum of 4 weeks, had repeat sonography to assess RVO and were subsequently followed for recurrent events. *Results*. Thirteen studies met inclusion criteria: 3531 patient VTE events with 3474 evaluable results were analyzed. The presence of RVO was associated with recurrence in all VTE (OR 1.93; 95% CI: 1.29, 2.89) and secondary VTE (OR 2.78; 95% CI: 1.41, 5.5) but not for primary VTE (OR 1.35; 95% CI: 0.87, 2.08). When cancer patients were eliminated from the secondary VTE group, there was no longer a significant association of RVO with VTE recurrence (OR 1.73; 95% CI: 0.81, 3.67) while in the subset of cancer patients, presence of RVO was associated with an increase in VTE recurrence risk (OR 5.14; 95% CI: 1.59, 16.65, *P* < 0.006). *Conclusions*. We conclude that the presence of RVO is associated with recurrence in secondary VTE but not in primary VTE and that association may be driven by the subset with cancer.

## 1. Background

Venous thromboembolism (VTE) is a common disease and long-term anticoagulation is effective in the prevention of recurrent deep venous thrombosis (DVT) and pulmonary embolism (PE). But anticoagulation is associated with bleeding complications necessitating a continuous assessment of bleeding risk versus recurrent thrombosis risk. Recent guidelines suggest that primary (unprovoked) VTE should be anticoagulated for 3 months and be evaluated for lifelong anticoagulation, whereas only 3-month anticoagulation is recommended for secondary (provoked) VTE [1–5]. In order to predict who will recur at the end of 3 months after a period of anticoagulation, clinical decision rules and laboratory surrogate markers have been developed. Current markers, however, are poor in predicting individual recurrence risk and better surrogate tests are needed [6–10]. One such test is using the presence of residual vein obstruction (RVO), after completing the period of anticoagulation, as demonstrating increased recurrence risk. However, various investigators have used different definitions for RVO [11, 12] and different studies assessing the predictability of RVO have yielded different results. These disparities may be due to the heterogeneity of studies, different patient populations, and/or the varying lengths of anticoagulation. In order to better understand these results, we performed an updated meta-analysis of the published studies to determine whether RVO after a period of anticoagulation can predict VTE recurrence risk in patients with primary or secondary VTE.

## 2. Methods

### 2.1. Data Source

A comprehensive literature search with the terms “residual vein thrombosis”, “residual vein obstruction”, and “recurrent venous thromboembolism” was performed on MEDLINE, EMBASE, Web of Science, and Science Direct. Articles in English between January 1990 and December 2011 were eligible for this analysis.

### 2.2. Study Selection

All abstracts were reviewed and selection was based on the following criteria: studies had to be prospective; the VTE should have been treated with anticoagulation for at least 4 weeks with unfractionated heparin, low molecular weight heparin, or warfarin; compression ultrasound (CUS) was performed to assess the presence of RVO; recurrent thromboembolic events at the cessation of anticoagulation were recorded.

RVO was defined by any of three criteria: Group (A): Prandoni criteria—if the transverse diameter was >2 mm at maximal compression [11]; Group (B): Siragusa criteria—residual thrombus greater than 40% of the vein diameter [12]; or Group (C): presence or absence of residual thrombosis or normal or abnormal Doppler scan. Recurrent events were defined as a confirmed new PE by perfusion scan, computed tomography angiogram, or pulmonary angiography; a new contralateral DVT; or a new ipsilateral DVT. Recurrent ipsilateral DVT was defined in all studies by demonstration of a newly noncompressible segment in a previously compressible vein. Additionally, some studies also defined recurrent VTE when thrombus extension of >2–4 mm was noted during CUS [12–19], when there was evidence of increased clot extension from ipsilateral ascending venography [20, 21] or in the presence of high clinical likelihood for DVT in the presence of thrombus extension when compared with a previous scan [22, 23]. In one study [24], the method of diagnosis of recurrent VTE was not specified and the author did not respond when contacted. Only a few studies standardized their measurement of the RVO by some form of video demonstration before the study.

### 2.3. Data Extraction

Three reviewers (Matthew Sullivan, Shuang Guo, and Marina Shcherba) independently assessed the studies and extracted the data (baseline characteristics of all studies, review of outcomes, and review of events) using a standardized data spreadsheet. Discrepancies were addressed and adjudicated by another independent reviewer (MJ).

### 2.4. Data Analyses

Data were analyzed with meta-analysis software developed by StatsDirect (StatsDirect Ltd. http://www.statsdirect.com/. England 2008). Odds ratios (OR) with 95% confidence intervals were calculated for individual studies and for pooled analysis, and Forrest plots were generated. Publication bias was assessed by the Horbold Egger test and by funnel plot. A priori the studies were considered heterogeneous due to different demographics of the population, different methods of RVO measurement, and the differing lengths of anticoagulation and heterogeneity was quantified by the *I* ^2^ statistic. Studies were not pooled if the *I* ^2^ was greater than 75% indicating significant heterogeneity. When studies were found to be moderately heterogeneous, the random effects model for calculation of OR's was used and reported.

## 3. Results

### 3.1. Study Selection and Characteristics

**Figure 1:**
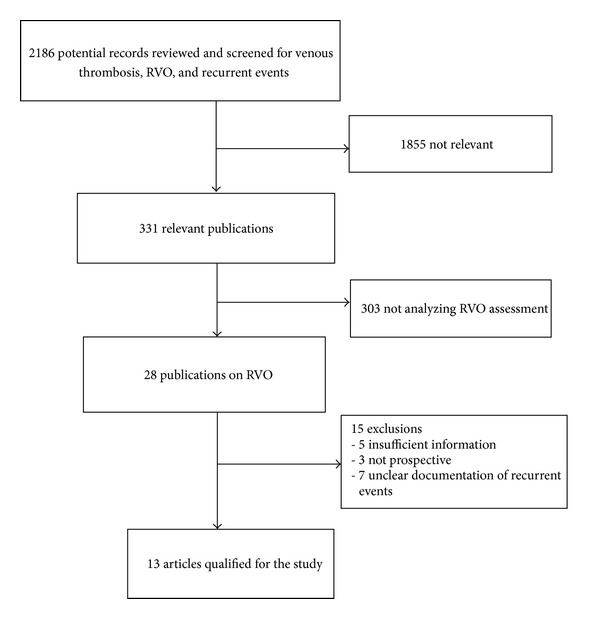
Flow diagram of study meta-analysis group selection.

We identified 2186 potential publications from the database search from which 28 were relevant to residual vein thrombosis and had recorded data on recurrent events of venous thrombosis (Figure 1). Thirteen studies [12–24] met inclusion criteria and were included in the study (Table 1). When assessed for publication bias by funnel plot, the unselected VTE group appeared to show some heterogeneity; analysis of the subgroups demonstrated primary VTE studies to be more homogeneous with some publication bias noted for studies analyzing secondary VTE. There was no publication bias as defined by the Horbold Egger test in the analysis for all VTE or when analyzed in subgroups for primary and secondary VTE.

Five studies recruited only patients with primary VTE, four studies looked at secondary VTE only, and three other studies looked at both primary and secondary VTE. In addition, one study had event rates for both primary and secondary VTE separately, and hence in subgroup analysis, this study was broken down to separate patients into their respective groups [17]. 

The thirteen included studies contained 3,531 patient VTE events of which 3474 could be evaluated by RVO assessment, across different countries, with a mean age of 61 years (Table 1). 2278 patients (64.5%) were classified as primary VTE; of these only 1874 patients could be identified as primary VTE with evaluable RVO studies. Similarly, there were 1253 secondary events of which 856 were defined as secondary with evaluable RVO studies. Two studies [18, 24] included only cancer patients, while other secondary VTE studies [17, 19, 21] excluded cancer patients. In three studies, the subgroups could not be differentiated for analysis [12, 22, 23]. The compression ultrasound was typically done on the day of stopping anticoagulation and the mean follow-up was 22 months after cessation of anticoagulation. RVO was present in 1712 patients (49.3%) of which 285 (16.6%) had recurrent VTE, while 177 of 1762 (10.1%) in the RVO negative group had recurrent VTE within their observation period. The minimum duration of anticoagulation was 3 months in all studies except in one where it was 4 weeks for secondary VTE [21]. 

**Figure 2:**
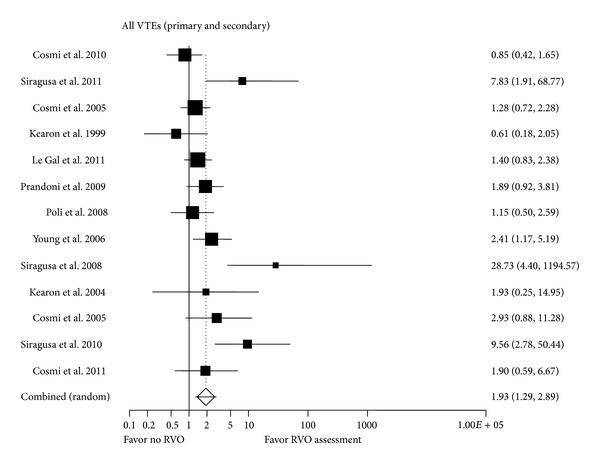
All VTEs. Forrest plot for RVO assessment.

**Figure 3:**
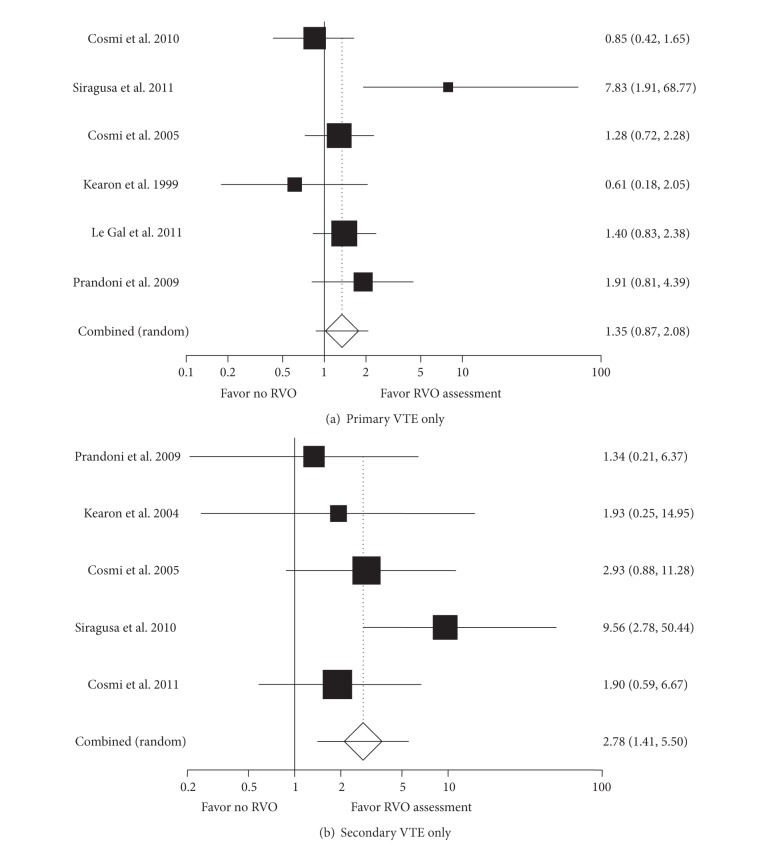
Meta-analysis for primary (a) and secondary (b) VTE.

**Figure 4:**
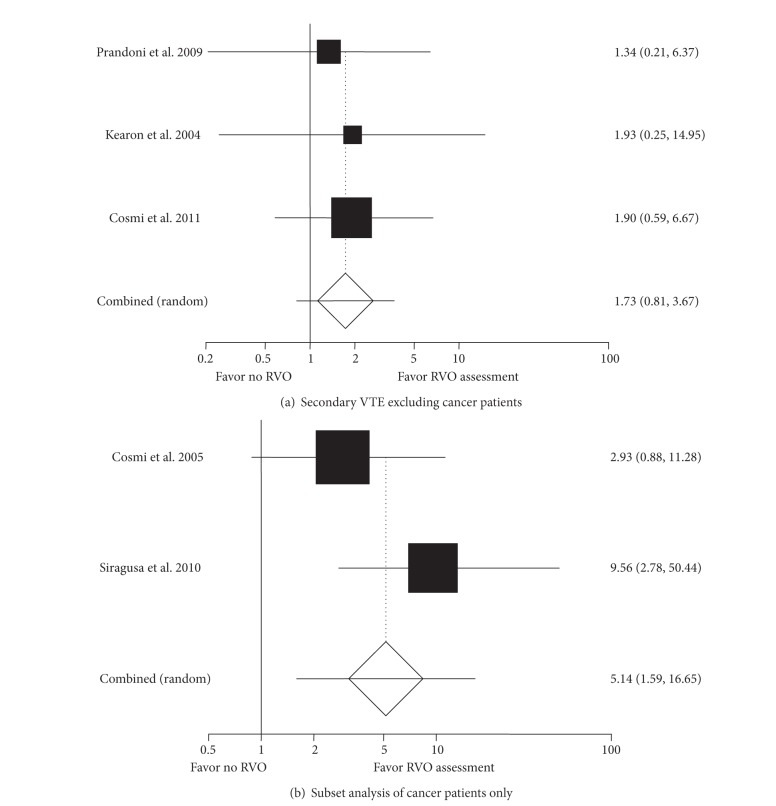
Subgroup analysis for secondary VTE, excluding cancer (a) and cancer patients only (b).

For the entire group with VTE (Figure 2), the presence of RVO was associated with a significantly higher recurrent VTE risk (OR 1.93, 95% CI: 1.29, 2.89, *I* ^2^ = 64%). For primary VTE alone (Figure 3(a)), RVO failed to demonstrate a statistically significant increased recurrent VTE risk (OR 1.35, 95% CI: 0.87, 2.08, *I* ^2^ = 54%). When results were analyzed only for patients with secondary VTE (Figure 3(b)), the presence of RVO was more strongly associated with an increased risk of VTE recurrence (OR 2.78, 95% CI: 1.41, 5.50, *I* ^2^ = 32%). When patients with cancer were eliminated from the secondary VTE cohort (Figure 4(a)), the OR decreased to 1.73 (95% CI: 0.81, 3.67, *I* ^2^ = 0%) and was no longer significant. However, for the two studies with cancer patients (Figure 4(b)), a positive RVO study was still significantly associated with VTE recurrence (OR 5.14 95% CI: 1.59, 16.65, *P* = 0.006).

The different methods of RVO assessment did not appear to have a differentiating effect. The risk of recurrent DVT given a positive RVO was significant when either the Prandoni (see Section 2 and Group A) or the non-Prandoni (see Section 2 and Groups B and C) measurements were used (OR 1.67, 95% CI: 1.02, 2.72, *I* ^2^ = 59.5% versus OR 2.36, 95% CI: 1.14, 4.89, *I* ^2^ = 70.6%, resp.).

When the mean anticoagulation period was <6 months, the risk of recurrent DVT given a positive RVO as compared to a negative RVO was 2.15 (95% CI: 1.02, 4.52, *I* ^2^ = 61%) and when >6 months, the odds ratio for rethrombosis was 1.85 (95% CI: 1.12, 3.06, *I* ^2^ = 70%).

## 4. Discussion

VTE is a chronic recurrent condition contributing to increasing morbidity and mortality. The 5-year cumulative incidence of recurrent venous thromboembolic events is 21%–28% [25–29]. The major impediment to long-term anticoagulation is the bleeding risk which must be balanced against the high risk of thromboembolism [20, 30, 31]. The optimal duration of therapy after 3 months in patients with primary VTE is currently unclear and the case for continued anticoagulation must significantly outweigh the bleeding risk. Markers to predict those with a higher thrombosis risk would help balance the risk for continued anticoagulation.

Residual venous obstruction (RVO) is currently defined as the persistent presence of clot as measured by compression Doppler ultrasonography at the site of the original DVT after some period of time. Studies have evaluated thrombus regression by CUS in patients with symptomatic deep vein thrombosis (DVT) of the lower limb. Normalization rates after a first episode of DVT range from 23% to 100% at 1 year [32–34]. We have demonstrated that the average clearance for the populations studied is approximately 50%. Large thrombus burden, younger age, immobilization, previous occurrence of recurrent episodes, DVT involving the entire femoral-popliteal veins, and duration of symptoms prior to treatment have been found to be unfavorable factors for normalization [32]. Thus, for example, 6 months after the acute DVT, C-US normalization was observed in 100% of postoperative patients versus 53% of cancer-free outpatients and in only 23% of outpatients with cancer [32].

The rationale behind studying RVO is that the rate of venous recanalization may be indicative of a systemic imbalance between thrombus propagation and fibrinolysis and that the presence of a RVO after a period of anticoagulation may reflect an ongoing systemic prothrombotic state or decreased fibrinolytic activation that puts the patient at higher risk of recurrent DVT [35, 36]. Our meta-analysis shows that the presence of RVO correlates with an increased risk of recurrence for all VTE but, when analyzed separately, not for patients with primary VTE. This is consistent with previous observations [37, 38]. Data for patients with secondary VTE do demonstrate an increased recurrence risk; when we excluded patients with cancer, the RVO studies were no longer predictive (*P* = 0.12). In our study, 7.3% (15/206) of patients with RVO and 4.8% (16/334) of patients with no RVO developed recurrent VTE in the subgroup with secondary VTE. The odds ratio for a positive RVO in this noncancer group is 1.73 (95% CI: 0.81, 3.67) and is actually higher than that in the group with primary VTE but is not statistically significant. However, the wide confidence intervals may suggest either that there may be a small subset of patients who do not clear their clot after a secondary VTE within a larger group who really are at a higher risk, that the numbers involved in the secondary group without cancer are too small (542 patients in the secondary noncancer group as opposed to 1877 in the primary group), or that it may indeed simply be nonsignificant. 

**Figure 5:**
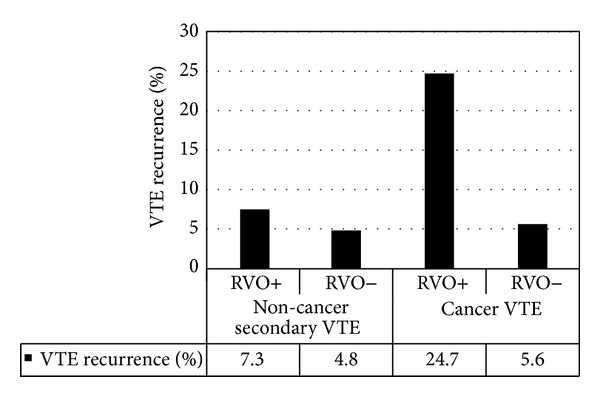
VTE recurrence in noncancer and cancer patients: association with RVO studies.

In the group with cancer, since there were only two studies, it is difficult to draw any definitive conclusions, but both studies showed a significant association of recurrent VTE with RVO. 24.7% (43/174) of patients with RVO and 5.6% (8/142) of patients without RVO developed VTE in the follow-up (Figure 5). In the study by Cosmi et al. [19], patients with metastatic cancer or requiring chemo- or radiotherapy were excluded, essentially limiting the patient population to limited stage disease, but even in this population, the risk of VTE was increased if they had RVO after 3 months of anticoagulation (OR 3.8, 95% CI: 1.11, 13.38, *P* = 0.033). In the second study by Siragusa et al., 24% had advanced cancer. Hence this group would have had indications for continued anticoagulation. This study was done to detect differences between continuing anticoagulation on the basis of RVO and they concluded, as our further evaluation supports, that the absence of RVO identifies a patient population with low risk of further VTE. This study may be confounded by the fact that there was a higher number of patients with advanced cancer in the RVO positive rather than the RVO negative group, as might be expected (*P* = 0.03). Still, combining these results suggests that larger studies need to be done in this subgroup of patients. Current ACCP and NCCN guidelines for thrombosis in cancer differ in their recommendation for duration of anticoagulation, but both recommend extended anticoagulant therapy. If even a small subset can be identified who do not require extended AC, it would be beneficial for the patients in terms of savings in time, inconvenience, side effects, and cost.

Since the studies were considered heterogeneous a priori, three sources of between-study heterogeneity were identified: (a) heterogeneity due to the cause of thrombosis—primary versus secondary; (b) heterogeneity due to various methods of measurement of RVO—Pradoni versus others; and (c) heterogeneity due to varying time periods of anticoagulation—less than 6 months versus greater than 6 months (mean time). Sensitivity analyses were done accordingly. When studies were analyzed according to the duration of anticoagulation or according to the method of diagnosis of RVO, there was a moderate heterogeneity between the studies. The average effect calculated by the random effects model suggests that RVO correlates with the recurrent risk of VTE despite different methods of diagnosis or varying lengths of anticoagulation. 

Our meta-analysis differs from previously published meta-analyses in that it also utilized studies which specifically included only secondary VTE. We suggest that it is primarily the secondary VTE that contributes to the overall observation that RVO can predict VTE recurrence.

The limitations in the meta-analysis include the sources of heterogeneity previously mentioned. Moreover there could be some publication bias as suggested by the funnel plot but not by the Horbold Egger test in the secondary VTE group. The relatively smaller number of patients in the subsets and the observational nature of the studies with cancer preclude any definitive conclusions from this study, which needs to be further explored. Our study poses further interesting questions—whether there is a subset of patients with secondary VTE in which RVO may be useful and whether RVO can predict a group of patients with cancer who do not need anticoagulation. These questions need to be answered in further prospective studies.

## Figures and Tables

**Table 1:** Characteristics of included studies.

Study	Design	Sites	f/u mos	Age	DAC (mo)	CUS date	STD	Criteria	Primary (*n*)	Secondary (*n*)	Proximal (*n*)	Distal (*n*)	n*	RVO+ (*n*)	RVO− (*n*)	RVO+ DVT+ (*n*)	RVO− DVT+ (*n*)
Cosmi et al. 2010 [8]	RCT	MC	18	63	≥3	SAC	Video	Prandoni	397	0	397	0	397	151	246	17	32
Siragusa et al. 2011 [14]	RCT	MC	12	55	3–24	3 mo	N/A	Siragusa	409	0	409	0	394	258	136	27	2
Cosmi et al. 2005 [15]	PC	SC	24	72	6	SAC	None	Prandoni	400	0	400	0	400	225	175	41	26
Kearon et al. 1999 [20]	RCT	N/A	24	59	3	SAC	None	+/−	83	0	83	0	81	46	35	8	9
Le Gal et al. 2011 [16]	PC	MC	17	54	5–7	SAC	Yes	+/−	452	0	452	0	451	231	220	45	32
Prandoni et al. 2009 [17]	RCT	MC	36	65	3	SAC	Video	Pradoni	151	117	268	0	268	79	189	19	27
Poli et al. 2008 [22]	PC	SC	25	62	≥3	SAC	None	Pradoni	183	112	295	0	258	105	153	14	18
Young et al. 2006 [23]	PC	SC	33	55	3–6	SAC	None	+/−	103	213	241	72	316	174	142	34	13
Siragusa et al. 2008 [12]	PC	MC	24	60	≥3	N/A	None	Pradoni	100	70	170	0	170	92	78	25	1
Kearon et al. 2004 [21]	RCT	N/A	12	56	≥1	SAC	None	+/−	0	129	88	41	129	45	84	3	3
Cosmi et al. 2011 [19]	PC	SC	24	60.8	5	SAC	N/A	Pradoni	0	296	296	0	294	132	162	9	6
Siragusa et al. 2010 [24]	RCT	N/A	12	59.3	6*	SAC	N/A	Siragusa	0	228	N/A	N/A	228	123	105	27	3
Cosmi et al. 2005 [15]	PC	N/A	24	71	≥3	SAC	N/A	Pradoni	0	88	88	0	88	51	37	16	5

RCT: randomized controlled trial.

PC: prospective cohort/open label.

MC: multicenter.

SC: single center.

+/−: present or absent, normal or abnormal.

STD: standardization method.

f/u: follow-up.

6*: LMWH given for the duration.

SAC: same day as stopping anticoagulation.

n*: total number eligible for analysis.

N/A: not available.

DAC: duration of anticoagulation.
